# Enhancing the Mechanical and Tribological Properties of Cellulose Nanocomposites with Aluminum Nanoadditives

**DOI:** 10.3390/polym12061246

**Published:** 2020-05-29

**Authors:** Shih-Chen Shi, Tao-Hsing Chen, Pramod Kumar Mandal

**Affiliations:** 1 Department of Mechanical Engineering, National Cheng Kung University (NCKU), Tainan 70101, Taiwan; n16047151@mail.ncku.edu.tw; 2 Department of Mechanical Engineering, National Kaohsiung University of Science and Technology, Kaohsiung 83301, Taiwan; thchen@nkust.edu.tw

**Keywords:** HPMC, Al, mechanical property, FTIR, tribology, bonding

## Abstract

Hydroxypropyl methylcellulose (HPMC) is a common hydrophilic and biodegradable polymer that can form films. This study incorporated aluminum nanoadditives as an enhancement reagent into a HPMC matrix. Mechanical properties of nanocompoistes, including the tensile strength and the elastic modulus, were analyzed with a nano-tensile tester. The incorporation of additives in HPMC films significantly enhances their mechanical and film barrier properties. Evidence of bonding between the additive and matrix was observed by Fourier-transform infrared spectrometer analysis. The additives occupy the spaces in the pores of the matrix, which increases the tendency of the pore to collapse and improves the chemical bonding between the base material and the additives. The incorporation of excess additives decreases the tensile strength due to ineffective collisions between the additives and the matrix. The wear test proves that the addition of nano-additives can improve the tribology performance of the HPMC composite while reducing the wear volume and the friction. Bonding between the nanoadditives and the matrix does not help release the nanoadditives into the wear interface as a third-body layer. The main reason to enhance the tribology performance is that the nanoadditives improve the load-capacity of the composite coating. This hybrid composite can be useful in many sustainability applications.

## 1. Introduction

Hydroxypropyl methylcellulose (HPMC) is the most commonly used hydrophilic, biodegradable polymer to form films, primarily because HPMC functions as a pH-independent and viscous gelling agent. It is the most important hydrophilic carrier material used to prepare orally controlled drug delivery systems, and it exhibits thermal gelation properties. In other words, when the solution is heated to a critical temperature, the solution congeals into a non-flowable but semi-flexible mass. This critical temperature is inversely related to the concentration of the HPMC in the solution and the concentration of the methoxy groups within the HPMC polymer. As the concentration of methoxy groups increases, the critical temperature decreases. The inflexibility/viscosity of the resulting mass is also directly related to the concentration of the methoxy groups (with a higher concentration, the resulting mass is more viscous and less flexible).

The efficacy of cellulose and its derivatives has been proposed as a possible alternative for non-degradable synthetic plastics for use in bio-based packaging applications. This bio-based polymer is an attractive alternative for existing packaging materials due to its biodegradability, renewability, and large-scale availability at a relatively low cost. Among the cellulose derivatives, HPMC is a good material for films, and it has potential for packaging applications due to its flexibility, transparency, and resistance to oil and fat [1]. Moreover, HPMC has already been approved for food-based applications, which makes it suitable for preparing edible films and coatings [2]. Recently, many studies on bio-based research topics have been proposed, which also show great potential for future applications, such as montmorillonite nanoclay [3] and bio-based coatings [4].

Several studies have examined the characterization of HPMC biopolymers with regard to their tribology properties [5,6,7,8,9], green protective layer [10,11], self-healing properties [12,13], corrosion inhibition [14,15], and quick and easy analysis [16,17]. Biopolymer materials display a good performance in a variety of fields; however, there remain some gaps with respect to the knowledge of fundamental biopolymer characteristics in comparison to synthetic polymers. Therefore, some studies have investigated the mechanism of biopolymer reinforcement [18,19,20,21], specifically in mechanical applications [22,23]. However, there are few basic studies on the HPMC matrix, such as the influence of its molecular weight on mechanical properties and its anti-wear properties.

Aluminum is the second most widely used metal in the world. It is desirable because of its low density, high strength, excellent corrosion resistance, and ease of recyclability. Aluminum is generally nontoxic, and it naturally occurs in many food products. The hydrophobic nature of aluminum makes nanometric aluminum attractive. Significant efforts have been made to modify the hydrophobicity of aluminum surfaces [24,25].

Previous studies have shown that the addition of particulate additives can enhance the tribology properties of HPMC [26]. However, the influence of additives on the mechanical properties of the composite film, the correlation between the mechanical and tribology properties, and its wear resistance properties remain unclear. In this study, the mechanical properties of HPMC biopolymer films with a variety of molecular weights were investigated. In addition, the mechanism of strengthening the material and improving the mechanical properties by adding aluminum nanoparticles is discussed. The mechanism of additive-reinforced composites is also discussed. Glass displays are widely used in daily life. Under light load conditions, high penetrability and wear protection requirements are very suitable for the application of the aluminum nanoadditive-reinforced HPMC composite.

## 2. Experimental Details

### 2.1. Film Preparation

Different types of HPMC (Pharmacoat 645, 606 and 615, Shin-Etsu, Tokyo, Japan), based on the molecular weight, were obtained. The specifications of the different HPMC powders are listed in Table 1. Aluminum nanoparticles have an average diameter of 110 nm and a spherical appearance. The aluminum nanoparticles were purchased from I-Mei Materials, Co., Ltd. (New Taipei City, Taiwan).

**Figure 1:**
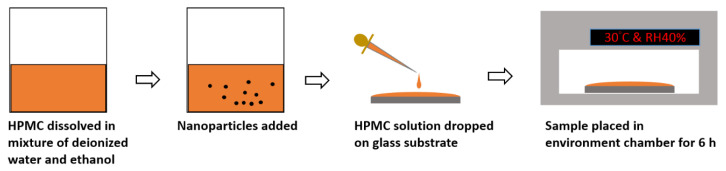
Schematic drawing of the film preparation process.

The HPMC powder (3 g) was added to a mixture of water and ethanol (20:80 mL) while performing magnetic stirring. Aluminum nanoadditives (113 ± 41 nm, spherical shape) with different concentrations were then added to the HPMC solution. Four concentrations of Al were used from 0 to 2 wt.%. The amounts of Al nanoadditives that were used in the film preparation process are shown in Table 2. The Al/HPMC solution was sonicated for 30 min and the mixture (30 mL) was poured into a glass substrate (Corning, Taiwan) and placed in an environmental chamber (DE60, Denyng Instruments, New Taipei city, Taiwan) at 30 ± 10 °C and RH 40% ± 10% for 6 h. In order to observe the effect of the interaction force between the additive and the HPMC matrix, no plasticizers were used for film formation. The film preparation process was demonstrated in Figure 1. 

### 2.2. Evaluation of the Mechanical Properties

The mechanical properties of the HPMC and the composite films were determined using a micro/nano tensile testing machine (DDS32, Kammrath and Weiss GmbH, 44141 Dortmund, Germany) while following the ASTM D882 standard. The thicknesses of the coatings were measured using a laser 3D profiler (VK9700, Keyence, Osaka, Japan). The coating thickness was controlled so that it was in the range 75 ± 5 μm.

### 2.3. Fourier Transform Infrared Spectroscopy (FTIR)

Attenuated total reflection mode of Fourier transform infrared spectroscopy (Thermo Nicolet NEXUS 470, GMI, Golden Valley, MN, USA) was performed to confirm the interfacial interaction between the additives and the HPMC matrix.

### 2.4. Tribology Behavior of the HPMC Composites

AISI 52100 chrome steel balls with a diameter of 6.35 mm were used as the counter bodies. Wear tests were conducted on a rotary ball-on-disk tribometer (POD-FM406-10NT, Fu Li Fong Precision Machine, Kaohsiung, Taiwan). The wear behavior under an applied normal force of 2 N and a sliding speed of 3 mm/s with a wear distance of 30 m was measured with dry sliding conditions. The tribometer was equipped with a controlled environment, i.e., a temperature of 25 ± 5 °C and a relative humidity of 70% ± 10%.

### 2.5. Third-Body Velocity Accommodation Mode

The relative speed of the two contacting objects is not zero. This indicates that the object moves relative to the maximum static friction force, which causes friction and wear [27,28]. The velocity accommodation mode is used to describe the movement state of the contact object and its interface at different relative speeds, with information on the sites and the manner in which the velocity is accommodated [29].

## 3. Results and Discussion

### 3.1. Mechanical Properties of the Nanocomposite Films

The mechanical properties of polymer materials are highly related to the molecular weight. In contrast, the tribology properties of soft film composites are strongly related to their strength and the characteristics of the additives [30]. This study first observed the mechanical properties of the HPMC matrix film with different molecular weights. Then, the matrix will form a composite with the aluminum additives to observe the effect on the mechanical properties and the tribology performance.

**Figure 2:**
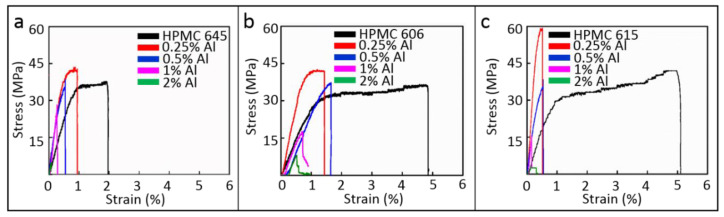
Nano-tensile tester results of (**a**) Al/hydroxypropyl methylcellulose (HPMC) 645 composite (**b**) Al/HPMC 606 composite (**c**) Al/HPMC 615 composite.

The tensile strength, elastic modulus, and nanocomposite elongation were measured to evaluate the mechanical properties of the composite films in comparison to pure HPMC. The stress–strain curves of the composite films with three different molecular bond lengths are illustrated in Figure 2. The results show that when the HPMC substrate molecular chain is long, the mechanical properties improve. By increasing the length of the HPMC matrix’s molecular chain, more intermolecular bonds will be produced between the chains. In addition, mechanical forces may exist between the long chains if they are entangled. Therefore, in comparison with the HPMC matrix, a higher molecular weight polymer (i.e., a polymer with a longer chain length) will provide excellent mechanical properties with a high elastic modulus (E) and a superb ultimate tensile strength (UTS). Adding a small amount of Al particles (0.25 wt.%) can increase the E and UTS; however, it simultaneously reduces the elongation (EL). The reason for this is that the additive may form a local bond with the matrix [31]. When more Al particles are added, the UTS and EL can decrease significantly. Too many additives may cause agglomeration, the characteristics of the high surface area may be lost, and defects may easily form, resulting in a reduction in the mechanical properties.

**Figure 3:**
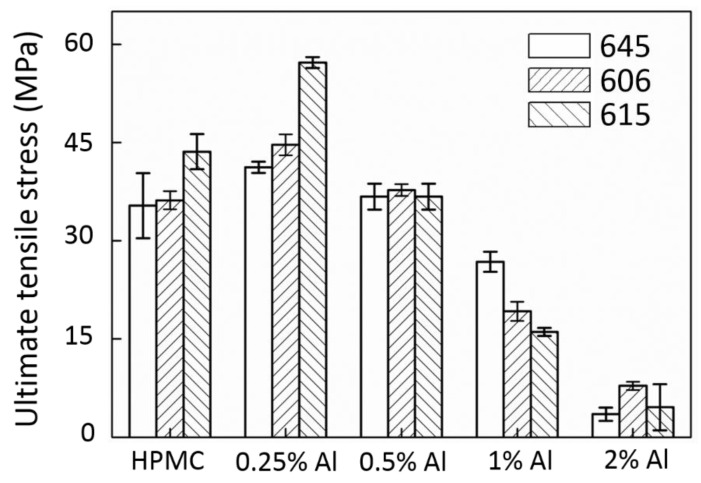
UTS results of the Al/HPMC composites.

**Figure 4:**
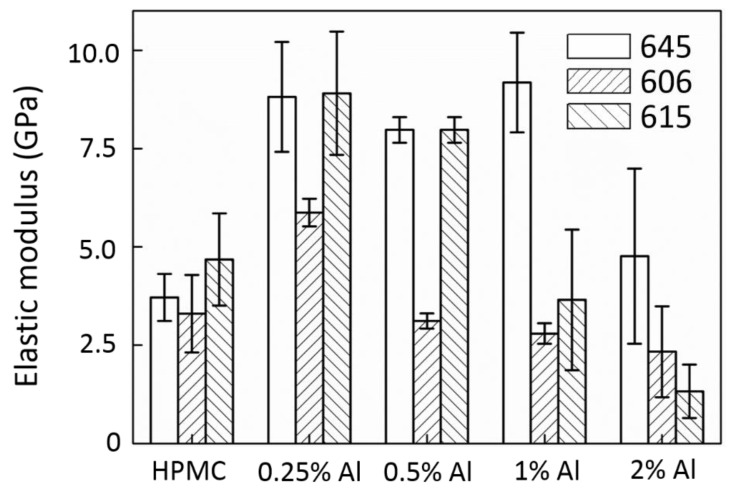
Elastic modulus of the Al/HPMC composites.

**Figure 5:**
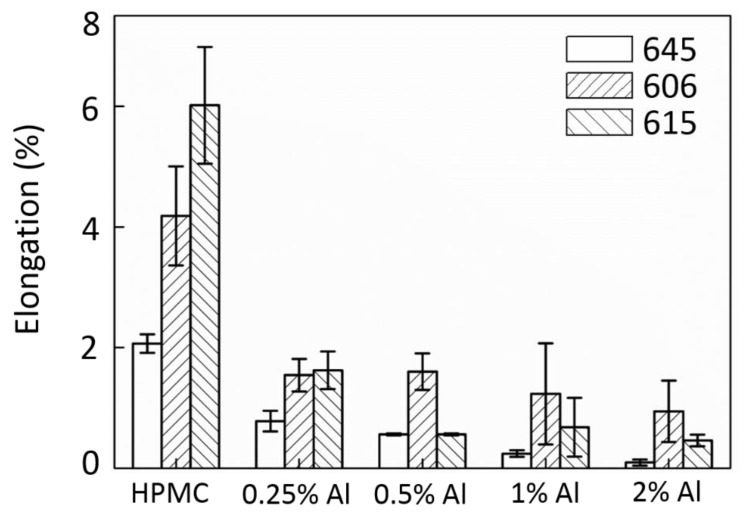
Elongation of the Al/HPMC composites.

The results from the tensile tests are further organized and plotted in Figure 3 (UTS), Figure 4 (E), and Figure 5 (EL). As displayed in Figure 3, the UTS of the composite film is best with a 0.25 wt.% Al addition. When the amount of incorporated Al increases, the UTS decreases significantly. The E of the composites is shown in Figure 4. Figure 4 demonstrates that a small number of additives can result in an enhancement effect. At this moment, the dispersibility of the additive plays an important role. The main factors that affect the particle dispersibility include the viscosity of the matrix solution and the size of the additive (agglomeration) [32]. In a low additive concentration condition, the dispersion of the additive is better, and the enhancement of the UTS and E is noticeable. When the additive concentration is high, it is easy to form an agglomeration due to its matrix viscosity, which causes a high fluctuation in the UTS and E. This phenomenon will be discussed in more detail in the subsequent FTIR analysis. The EL results in Figure 5 show that the addition of the Al nanoparticles can cause the film to become more brittle, thus, transforming them from materials that were previously elastic. However, in the application of anti-wear protective layers, a high specific strength and E are the main considerations.

According to Olea-Mejia et al., nanoparticle additives are better than micron-sized additives in terms of improving the tribology properties [33]. The main wear mechanism for the composite is adhesion after the polymer material and the nano-aluminum additive are mixed. The wear debris resembles sheet-like pieces of nano-aluminum metal that have been flattened. The sheet-like nano-aluminum metal coats are the first to wear, which can effectively reduce the wear rate of the substrate. In this study, nano-sized aluminum particles were added to embrittle the material properties of the Al/HPMC nanocomposite. Under applied pressure, when abrasion occurs, the metal on the surface of the nanocomposite can easily break down and leave the substrate. This will expose the Al particles and bring them into contact with the abrasive components to produce a sheet-like protective layer under the compressive stress, thus resulting in substrate wear reduction.

### 3.2. FTIR Evidence of Intermolecular Bonding

**Figure 6:**
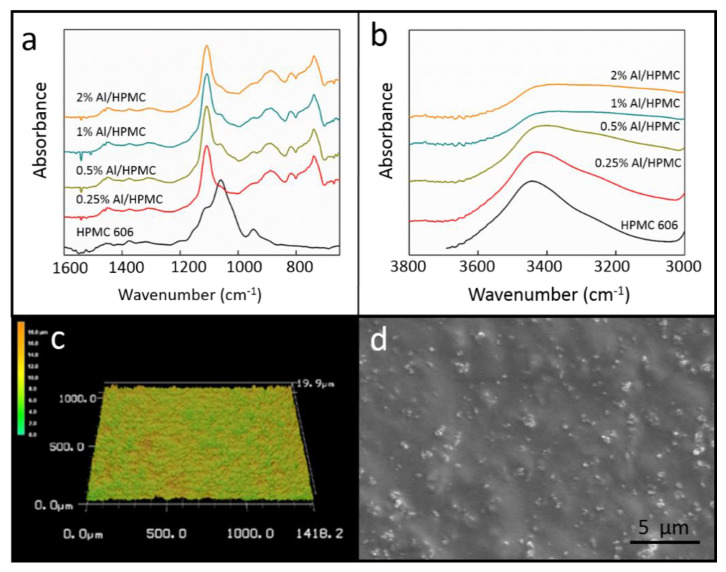
(**a**) FTIR spectra of the HPMC 606 composites with wave number 600-1600 cm^−1^ (**b**) FTIR spectra of the HPMC 606 composites with wave number 3000-3800 cm^−1^ (**c**) Surface pattern of Al/HPMC 606 (d) SEM image of Al/HPMC 606.

To further investigate the effects of Al nanoparticle additives on the interfacial bonding of the HPMC matrix, FTIR analysis was used to measure the changes in the bonding after particle addition. The FTIR spectra of Al/HPMC 606 composites are displayed in Figure 6. A new band was observed at 875 cm^−1^ after the addition of Al to the pure HPMC, which corresponds to the Al-O-OH group (Figure 6a). This depends on the interaction between Al^3+^ and the OH group of the HPMC. The peak observed at 750 cm^−1^ was due to the stretching vibration of Al-O in Al-O-OH (Figure 6a) [34,35]. However, intermolecular OH bonding of the composite film decreased when the amount of additive was increased, which resulted in a bond between Al^3+^ and OH^−^ (Figure 6b) [36]. Hence, the bands became broader and moved toward a lower wave number with increasing additive content. The results of the FTIR analysis show that the interfacial bonding decreases due to the addition of Al nanoparticles. This is consistent with the previous results shown by Figure 3, Figure 4 and Figure 5 in terms of the mechanical properties of the films. The surface pattern and SEM image of Al 1 wt.%/HPMC composite are shown in Figure 6c,d. The image shows that the additive and HPMC matrix are in good condition, and there is no obvious particle agglomeration.

### 3.3. Tribology Behavior of the Al/HPMC Composites

There is no significant difference between HPMC 606 and 615 in terms of their mechanical properties. HPMC 606 has a lower viscosity, which is better for the manufacturing process. Therefore, HPMC 606 was used as a matrix for the following wear test.

**Figure 7:**
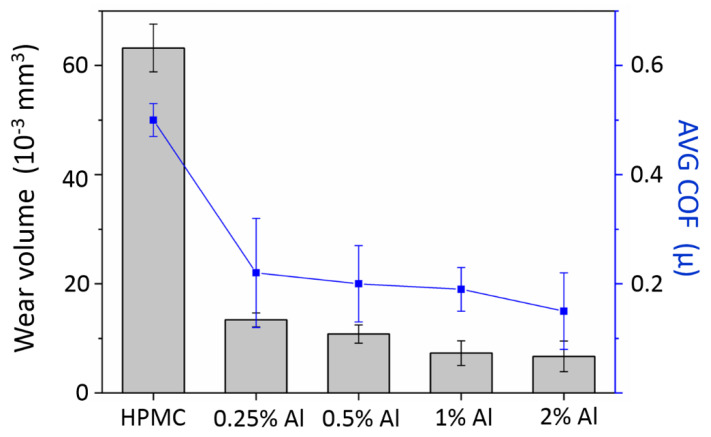
Tribology performance of the Al/HPMC 606 composites.

As shown in Figure 7, the addition of aluminum nanoadditives effectively reduces the wear volume by more than 70% and the friction coefficient by 60%. This shows that the contribution of the aluminum nanoadditives to enhancing the tribology performance of the composite is greater than the effect of the HPMC matrix. By comparing an additional amount of aluminum nanoadditives, a higher concentration of nanoadditives only slightly reduces the wear volume and the coefficient of friction. This shows that the increase in the number of nanoparticle additives only provides a higher load capacity. Based on the FTIR results of Figure 6, there is a bond between the Al particles and the HPMC matrix. The aluminum nanoadditives are not released to the interface of the wear pair during the abrasion process: they are trapped in the matrix. Additives play an important role during abrasion due to the need for speed accommodation. At this time, when the additive forms a bond with the polymer material, the additive cannot easily escape from the constraints of the matrix or provide a third-body wear mechanism to the system. It is very important to choose a proper concentration of additives [31]. A composite with a very high strength or one that is too brittle is not suitable for a dry abrasive layer. Therefore, in order to control the composite properties of dry lubrication and the protective coatings, proper strength, and toughness are important factors. In addition, bonding between the additive and the matrix must be controlled. A good composite material can provide a better load capacity. During abrasion, the additive particles can be released to the interface of the abrasion pair to form an efficient velocity accommodation mode, thus providing an effective wear mechanism.

## 4. Conclusions

Al/HPMC composite films were successfully prepared by the solvent evaporation method. Their mechanical properties, such as tensile stress and elastic modulus, were enhanced with the addition of 0.25 wt.% Al additive. The FTIR results revealed an interfacial adhesion between the Al nanoadditives and the HPMC matrix. The wear resistance and reduction in the coefficient of friction can be observed in the Al/HPMC composite. Aluminum nanoadditives provide an effective load capacity and good tribology performance. The lightweight and high specific strength dry wear protective layer that was prepared has the characteristics of sustainability, being easy to recycle and prepare with lubricating behavior. This can serve as a good candidate to be applied as a protective coating for large glass screens.

## Figures and Tables

**Table 1:** Specification of the HPMC powders.

Grade	Molecular Weight (g/mol)	Viscosity (mPa·s) @ 20 °C, 2 wt.%
HPMC 645	20,000	4.5
HPMC 606	35,600	6
HPMC 615	60,000	15

**Table 2:** Amount of Al nanoparticle additives and the corresponding weight percentages.

Al (g)	0	0.259	0.518	1.041	2.103
Al (wt.%)	0	0.25	0.5	1	2
